# Supplementary material for: A set of multi-entry identification keys to African frugivorous flies (Diptera, Tephritidae)
Source: Zookeys. 2014 Jul 24;(428):97–108. doi: 10.3897/zookeys.428.7366 (PMC4143993; doi:10.3897/zookeys.428.7366)
Supplement: Supplementary material 4 — Key to Capparimyia [file zookeys-428-097-s004.zip › SF4_ZooKeys_key to Capparimyia/lucid_player/help/help/using_the_player.htm]

Using the Lucid3 Player


# Using the Lucid3 Player

The Lucid3 Player has four main panels, a button bar and a menu (note:
the menu and button bar differ slightly between the Applet and Application
Players; the Application Player is shown below)

The four main panels display lists or trees of features and entities:

Features Available panel

Features Chosen panel

Entities Remaining panel

Entities Discarded panel

*Features
Available shows the features and states in the key. An identification
is performed by clicking on (and so ticking) score boxes alongside those
states that match the specimen being identified.*

In some keys, features and their states will be illustrated by images
displayed next to the feature or state name. See the topic Multimedia
in the Lucid3 Player for more information.

*Features Available* may display
features arranged as a list or as a hierarchical tree. See the topic Trees and Lists in Lucid3 for
more information.

*Features Chosen* shows the features
and states that have been used to describe the specimen being identified.

When an identification is started this window will be empty; as features
are progressively selected in *Features
Available*, they are copied to *Features
Chosen* for handy reference.

To unchoose a previously chosen state, click on (and so un-tick) its
score box in *Features Chosen*.

*Entities
Remaining* shows all entities that match the chosen features and
states.

In some keys, entities will be illustrated by images and web pages displayed
next to the entity name. In addition, a tab at the bottom of the Entities
panels provides access to a gallery of images for all remaining entities.
See the topic Multimedia
in the Lucid3 Player for more information.

*Entities Remaining* may display
features arranged as a ranked list or as a hierarchical tree. See the
topic Trees and Lists in Lucid3
 for more
information.

*Entities Discarded* shows all
entities that do not match the chosen features and states.

The button bar and menu contains many options to help make an identification
easier. See the topic The
Lucid Player�s Menu and Button Bar for links to help on these.
